# Anesthetic Approach for a Patient with Jeune Syndrome

**DOI:** 10.1155/2015/509196

**Published:** 2015-08-23

**Authors:** Mehmet I. Buget, Emine Ozkan, Ipek S. Edipoglu, Suleyman Kucukay

**Affiliations:** ^1^Department of Anesthesiology, Istanbul Medical Faculty, Istanbul University, Fatih, 34093 Istanbul, Turkey; ^2^Department of Anesthesiology, Suleymaniye Obstetrics & Pediatrics Training and Research Hospital, Zeytinburnu, 34116 Istanbul, Turkey

## Abstract

Jeune syndrome (JS) is an autosomal recessive disease also known as asphyxiating thoracic dystrophy. A narrow bell-shaped thoracic wall and short extremities are the most typical features of the syndrome. Prognosis in JS depends on the severity of the pulmonary hypoplasia caused by the chest wall deformity. Most patient deaths are due to respiratory problems at early ages. Herein, we report a case of JS patient, who was scheduled for femoral extension under general anesthesia. The severity of respiratory problems in JS patients is thought to diminish with age. Our case supported this theory, and we managed the anesthetic process uneventfully.

## 1. Introduction

Jeune syndrome (JS) was defined by Jeune et al. [1] in 1955 and is a rare form of skeletal dysplasia with an incidence of 1 in every 100,00–130,000 live births [2]. Jeune syndrome patients typically present with a narrow bell-shaped rib cage, a distended abdomen, and short arms and legs. Radiological examination often shows narrow chest cage, short and horizontal ribs, bicycle handlebar-shaped clavicle, pronounced costochondral junctions, short extremities, hypoplastic pelvic iliac wings, and acetabular bone protrusions (trident acetabulum) [3–5]. While the main characteristics of this syndrome are chest wall deformities and short extremities, renal, hepatic, pancreatic, and ocular involvements are also common [3, 6]. The structure of the thorax results in serious respiratory problems for JS patients [3]. During early childhood, they often die owing to respiratory distress. The severity of the thoracic deformity is the main prognostic factor [4, 7]. Infants with mild JS often present with recurrent respiratory infections. Renal and hepatic involvements are common among patients who survive the early childhood manifestations of JS, and these involvements often determine the prognosis [7, 8].

In this report, we present a case of JS in a 5-year-old boy who was scheduled for femur-lengthening surgery.

## 2. Case Description

We obtained written and oral informed consent from the patient's parents for the publication of the report. The patient was a 5-year-old boy born at term (weight: 3140 g; height: 47 cm). After birth, he was mechanically ventilated for 15 days and placed in the intensive care unit (ICU) for 23 days. He had tuberculosis at the age of 2 years and was prescribed antituberculosis drugs for 6 months. Until the age of 3 years, he had recurrent respiratory infections and underwent frequent hospitalizations. One year prior to this presentation, he underwent surgery for polydactyly and was in the ICU for a day.

**Figure 1:**
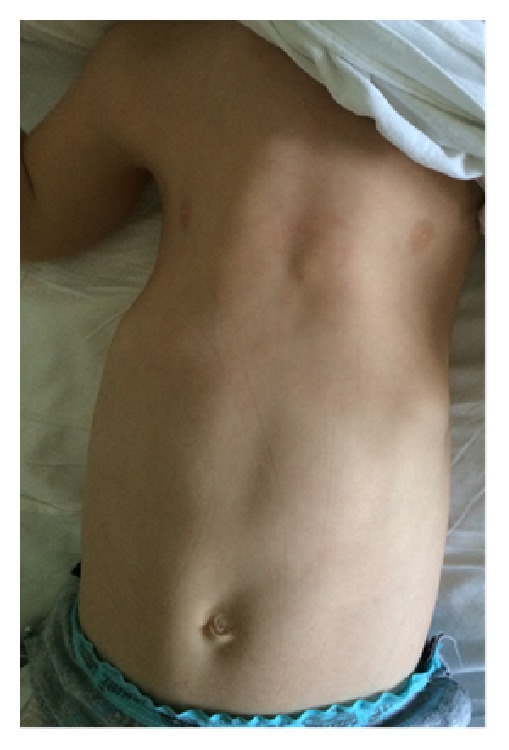
Narrow, long rib cage.

**Figure 2:**
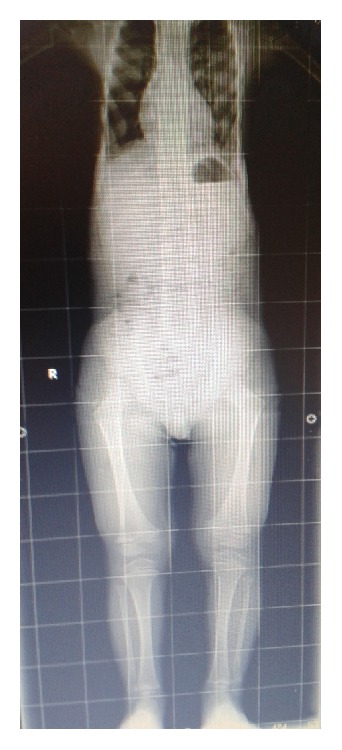
Radiography of patient. A long and narrow thorax, short and horizontal costae, and short extremities.

His present medical examination showed height and weight of 85 cm and 16 kg, respectively, and a normal mental status. He had a long, narrow chest wall and bilateral short extremities (Figure 1). His respiratory examination showed normal results. Renal, hepatic, and ocular functions also were normal. Radiologic presentation showed a long and narrow thorax, short and horizontal costae, and bicycle handlebar-shaped clavicles (Figure 2).

Pulse oximetry and electrocardiogram (ECG) were performed, and arterial blood pressure was monitored. We preferred to use an inhalational agent for anesthetic induction, because his Mallampati score was 1. We induced general anesthesia with 8% sevoflurane and mask ventilation. Once the mask ventilation was successful, we placed an intravenous catheter for fentanyl (2 *μ*g/kg) and rocuronium (0,6 mg/kg) administration. We intubated the trachea with a 5.0 cuffed endotracheal tube. We did not detect any problems during the intubation. We preferred pressure-controlled mechanical ventilation for our patient. The anesthesia was maintained with 2% sevoflurane, 40% oxygen, and 60% nitrogen. The operation was uneventful. The perioperative ventilation parameters were as follows: 20 mmHg peak pressure and 110 mL tidal volume with a frequency of 18/min. His arterial blood gas analyses showed the following: pH, 7,38; pO_2_, 164 mmHg; pCO_2_, 42 mmHg; and oxygen saturation, 99%. Length of the operation was 135 minutes. We did not use muscle relaxation monitor for our patient because to our knowledge we could not detect any association between JS and delayed recovery from muscle relaxants in the literature. The patient's trachea was successfully extubated after we ensured sufficient respiratory efforts. He was under observation for an hour in the postanesthesia care unit (PACU). We did not detect any respiratory problem or desaturation events. He was transferred to the ward after his modified Aldrete Score was 10.

## 3. Discussion

Jeune syndrome is one of the ciliopathies, and diagnosis is mostly based on clinical and radiological findings [4, 7]. Cilia are microtubule structures that support cell proliferation and differentiation, neural development, and tissue integrity. In ciliary disorders, multiple tissues and organs are often involved [9]. In our case, genetic analysis of the TCTEX1D2 gene, which is associated with JS and ciliopathies, revealed a homozygotic change [10, 11]. In the antenatal period, skeletal changes characteristic of JS can be identified by ultrasonography (USG) and subsequently diagnosed with genetic amniocentesis [12, 13]. Our patient was similarly diagnosed during the pregnancy.

Most JS patients die because of respiratory problems caused by chest deformities in early childhood. As the severity of the chest wall deformity is important for patient prognosis and because JS patients are often treated for recurrent respiratory infections preoperative anesthetic examination should be thorough and clinicians should be vigilant during respiratory examination. A chest radiograph, laboratory tests, and physical examinations should be performed. If a respiratory impairment is detected, blood gas analysis and spirometry are also advisable. In the preoperative settings, JS patients' lung volumes are reduced due to pulmonary hypoplasia, and, with positive-pressure ventilation, barotrauma and pneumothorax may occur [14, 15]. Additionally, increased thoracic pressure can decrease venous return and diminish cardiac output [2]. For these reasons, JS patients' ventilation settings and regular follow-ups are very important. The lowest possible peak pressure ventilation should be provided. Hence, Saletti et al. suggested pressure-controlled ventilation and Şahin et al. suggested low tidal volume (7 mL/kg and 16/min frequency) strategies in their reports [2, 14]. Both strategies were applicable to our case. We used pressure-controlled ventilation and the lowest possible peak pressure, and we managed to avoid any barotrauma risks.

Different surgical techniques are available to facilitate chest wall volume increases in the management of JS, although the success of the treatment depends on the level of the pulmonary hypoplasia [3, 16].

De Vries et al., in their case series involving 13 patients, proposed that JS patients who survive early childhood present with relatively diminished respiratory problems later in life [3]. They reported that 11 of their 13 patients had milder respiratory problems at older ages than they did in early childhood, possibly because the mechanical properties of the thorax get sufficiently altered with growth [3]. Our case supports this hypothesis. In our case, artificial respiration was needed for postpartum respiratory failure resulting from pulmonary hypoplasia. After 1 month of respiratory support, intensive care was no longer needed. Until 3 years of age, he was hospitalized for intermittent respiratory infections, for which he received treatments. At the age of 5, he underwent the surgical procedure described herein, without any serious adverse events. We acknowledge that an absolute conclusion about the diminishing of JS-related respiratory problems with age is not possible, noting that Leroy et al. presented a case of JS in a 13-year-old patient with respiratory problems [17].

Thirty percent of JS patients are thought to have renal insufficiency [3]. Kidney involvement, which can be detected by clinical and objective findings, typically would be seen after 2 years of age [3]. In our case, recent kidney function tests and radiological studies were unremarkable. In JS, complications characterized by increases in transaminase levels, hepatomegaly, liver involvement, and pancreatic insufficiency, sometimes resulting in portal hypertension, can occur [3, 6]. In our patient, there were no clinical and radiological findings of any liver and pancreatic insufficiency. Jeune syndrome patients can show various kinds of ocular involvement such as retinitis pigmentosa. We did not detect any eye problems in our patient.

We suggest a thorough anesthetic profiling for patients with JS, because the disorder can involve different organs. Respiratory evaluation is of utmost importance for these patients, as it can have fatal consequences, especially in younger patients. We believe that patients with JS would be better suited for comprehensive surgeries at older ages than at early childhood given that respiratory complications can diminish with age in most cases.
